# Simultaneous Bilateral Rupture of Patellar Tendons in a Young Man Without Predisposing Factors or Systemic Disease

**DOI:** 10.7759/cureus.5469

**Published:** 2019-08-23

**Authors:** Kyle L Barner, Caitlin Gluck, Kevin A Witte

**Affiliations:** 1 Orthopedics, Kansas City University of Medicine and Biosciences, Kansas City, USA; 2 Emergency Medicine, Kansas City University of Medicine and Biosciences, Kansas City, USA; 3 Orthopedics, Saint Luke's Health System, Kansas City, USA

**Keywords:** patellar ligament, patellar tendon, extensor mechanism, rupture, spontaneous, tendon injuries, trauma, orthopedics, knee

## Abstract

Bilateral patellar tendon (BPT) rupture is an exceedingly rare cause of extensor mechanism failure with many patients having predisposing factors such as systemic disease, steroid, or antibiotic use that increase the risk. Patients may present with an inability to extend the knee, diffuse pain and swelling, and a high riding patella on lateral radiographs. We present a case of a young man without predisposing factors or systemic disease who experienced a BPT rupture while playing sports.

## Introduction

The complexity of the knee joint is unparalleled by any joint in the body. Common injuries and pathologies that affect the knee include ligament and meniscus tears as a consequence of trauma to the knee from a blow, twist, or fall and degenerative joint disease in the elderly population. The common extensor mechanism of the knee includes the quadriceps femoris muscles, the quadriceps tendon, patella, and patellar tendon. The quadriceps tendon receives contributions from the rectus femoris superficially, the vastus lateralis and medialis in the middle, and the vastus intermedius deep giving the tendon a trilaminar structure. Fibers from the superficial rectus femoris muscle continue over the anterior surface of the patella and become thickened inferior to the patella to become the patellar tendon before inserting onto the tibial tuberosity [1]. When this extensor mechanism is disrupted, patients lose the ability to extend their knee and functionality is lost leaving the patient disabled. While unilateral extensor tendon rupture is a rarity, bilateral ruptures are exceedingly uncommon. Currently, there are approximately 50 reported cases of bilateral patellar tendon (BPT) ruptures that have been published in the scientific literature and only a small minority in patients without systemic disease or risk factors such as systemic lupus erythematosus (SLE), chronic kidney disease, fluoroquinolone antibiotic or corticosteroid use [2]. Older patients (over 60 years) predominantly rupture the quadriceps tendon and younger, athletic patients (under 40 years) predominantly rupture the patellar tendon due to sports injuries [3]. Given the rarity, rupture can often be misdiagnosed leading to delayed treatment and potentially diminished future function of the joint [4]. This case highlights the possibility of rupturing both patellar tendons simultaneously in a healthy, young patient so that the diagnosis is considered when patients present with history, signs, and symptoms that may warrant an alternative diagnosis.

## Case presentation

A 27-year-old African American male with no personal or family medical history, no recent antibiotic, steroid, or medication use presented to the emergency department via ambulance with bilateral knee pain and an inability to ambulate. While playing basketball on his wet, flat driveway, he slipped with his right knee in a flexed position. Trying to catch himself from falling backward, he noted immediate pain and a tearing sensation in his right anterior knee. Still trying to prevent the fall, he shifted his weight to his now flexed left knee and felt another "pop" and pain. His knees buckled, and he fell to the ground with both knees in a flexed position. He was unable to stand or extend his legs at the knee joint.

On physical examination, the patient’s weight was recorded as 127 kilograms with a body mass index (BMI) of 39. He was lying supine in the emergency room bed with both legs fully extended. He stated that his pain was well-controlled with morphine sulfate and hydromorphone and he was not in distress at the time of examination. The superior portion of his knees showed fullness with loss of the typical anatomical landmarks for the patella. There was diffuse, mild swelling over both knees with bilateral patellar tenderness on palpation and a gap below the inferior poles with deep palpation. The patient was unable to straight leg raise or actively extend either knee, and passive range of motion elicited too much pain to attempt. The collateral ligaments and cruciate ligaments were intact with normal laxity. Additionally, there was no concern of neurovascular compromise.

**Figure 1:**
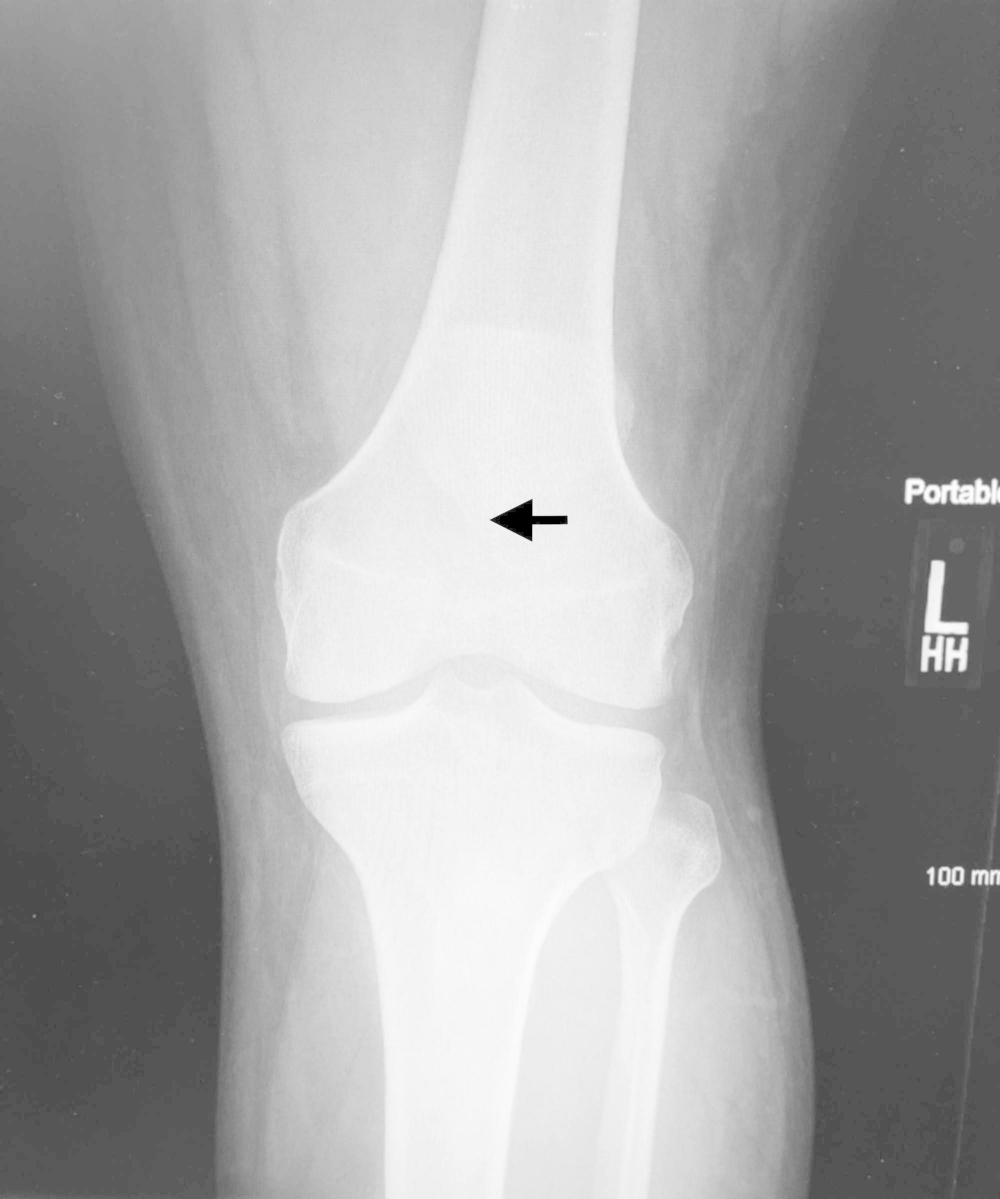
Anterior-posterior view radiograph of the left knee The apex of the patella is indicated (black arrow) demonstrating a high riding patella (patella alta).

**Figure 2:**
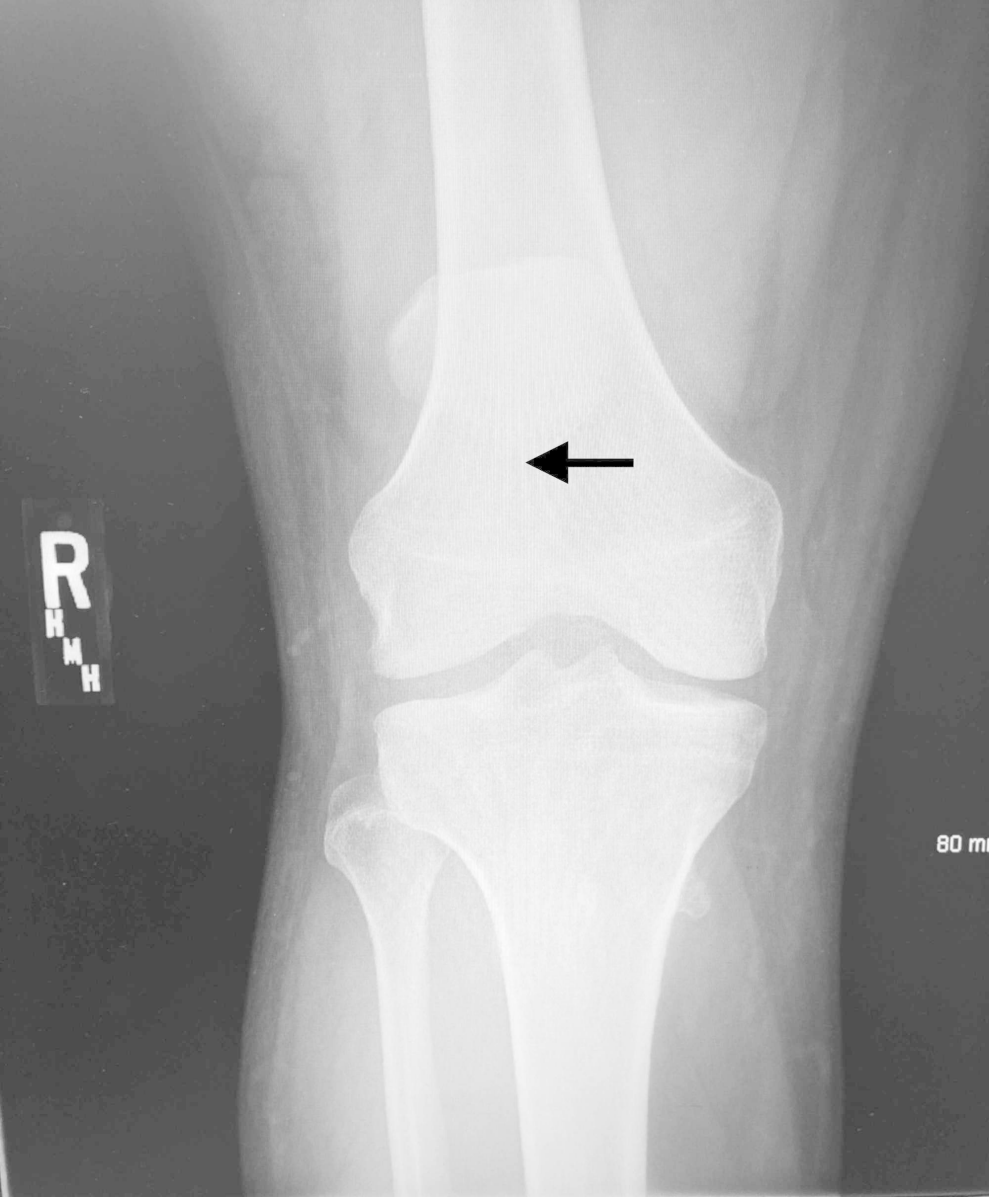
Anterior-posterior view radiograph of the right knee The apex of the patella is indicated (black arrow) demonstrating a high riding patella (patella alta).

**Figure 3:**
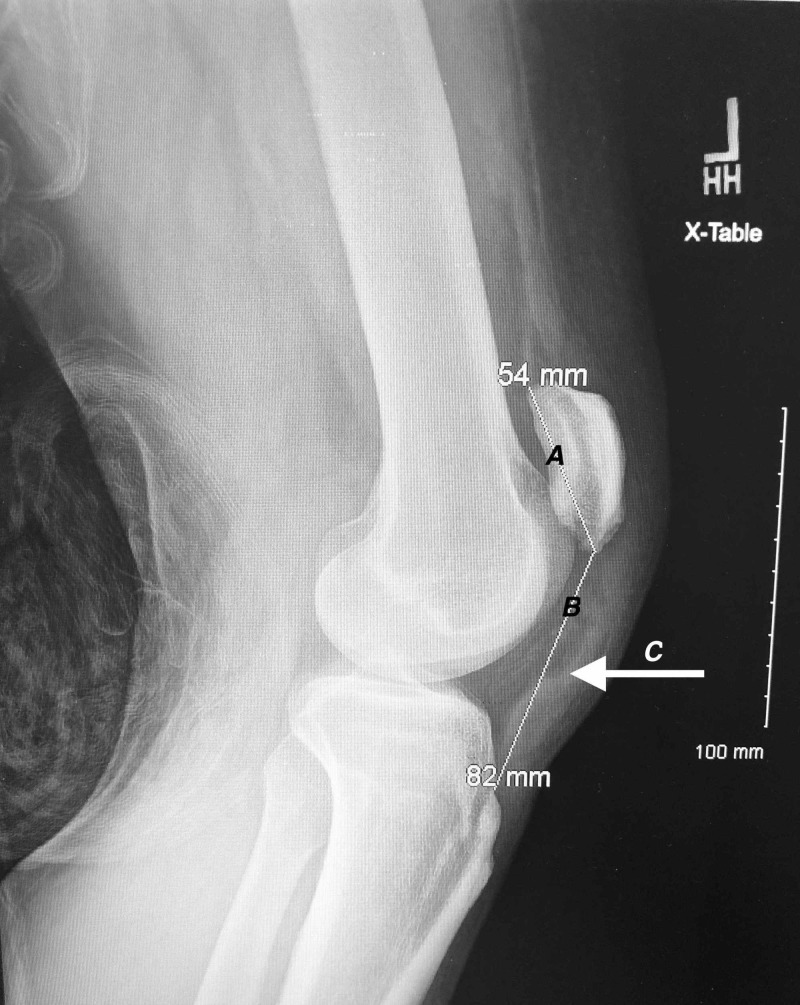
Lateral view radiograph of the left knee Line A indicates the patellar length of 54 mm measured from the superior pole to the inferior pole. Line B indicates the patellar tendon length of 82 mm measured from the lower pole of the patella to its insertion on top of the tibial tubercle. Arrow C indicates the position of the ruptured patellar tendon.

**Figure 4:**
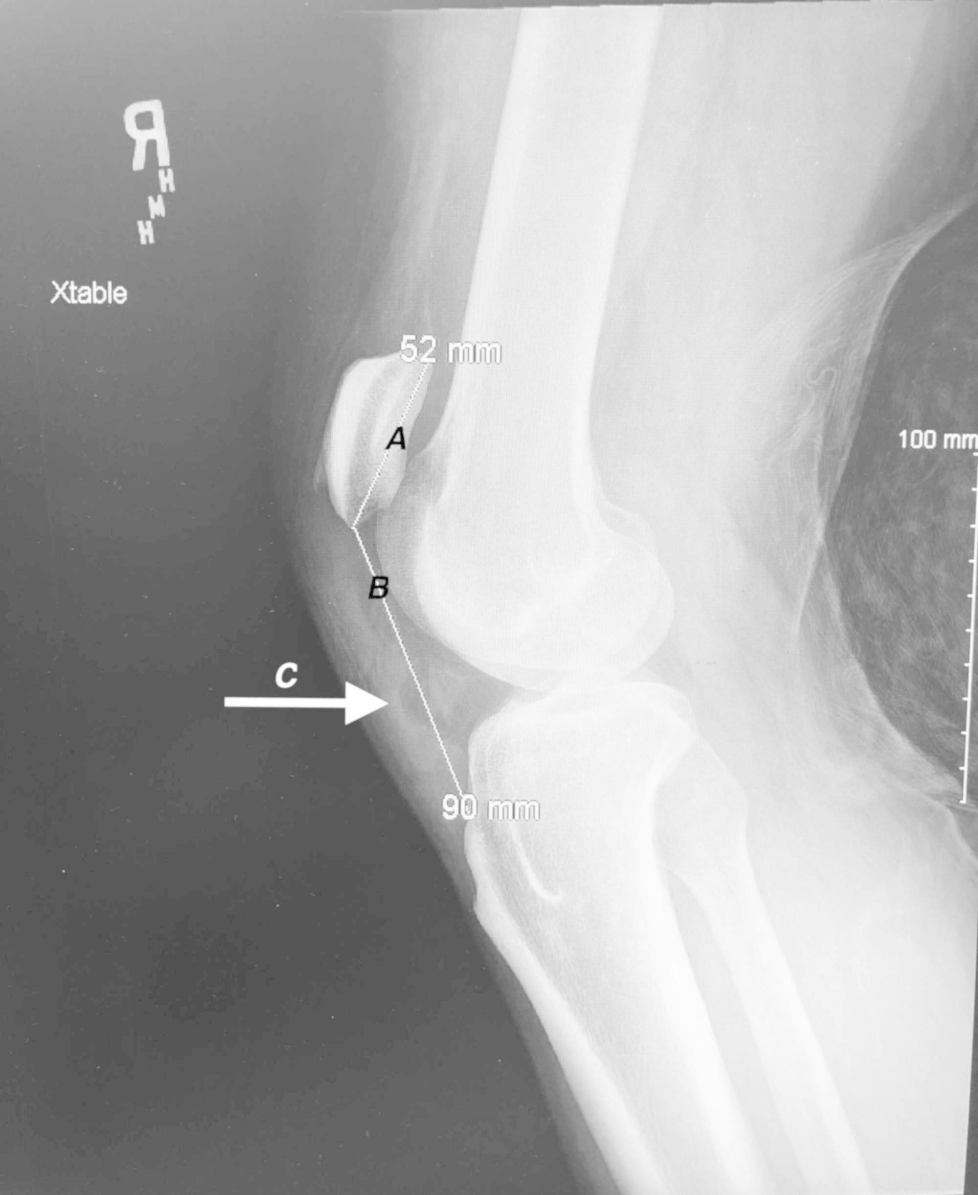
Lateral view radiograph of the right knee Line A indicates the patellar length of 52 mm measured from the superior pole to the inferior pole. Line B indicates the patellar tendon length of 90 mm measured from the lower pole of the patella to its insertion on top of the tibial tubercle. Arrow C indicates the position of the ruptured patellar tendon.

Plain radiographs of his knees revealed bilateral high-riding patellas (patella alta) on both anteroposterior and lateral films. Insall-Salvati ratios measured 0.66 and 0.58 for left and right knees, respectively (normal between 0.8 and 1.2 with values less than 0.8 indicating patella alta). Irregularity and inconsistency of the patellar tendons were consistent with rupture from the inferior pole of bilateral patellas (Figures 1-4).

The orthopedic surgery service was consulted, and he was admitted to the hospital. The following day, the patient underwent open repair of bilateral inferior pole patellar tendon ruptures and reconstruction of the tendons. A longitudinal midline incision directly over the normal anatomic location of the patella was made. Consistent with imaging, the patellar tendon was separated from the patella at the inferior pole bilaterally which is the most common site of rupture [5]. Additionally, extensive fraying of BPT was seen. The free ends of the tendon were located and debrided, and three double-loaded Arthrex BioComposite Swivelock Suture Anchors (Arthrex Inc., Naples, FL) (size 4.75) were utilized to reapproximate the tendon and reattach to the inferior pole of the patella. Collateral and cruciate ligaments were inspected for damage but were intact. The patient was placed in extension braces in the operating room, and the patient was advised against weight-bearing for two weeks. The remainder of his hospitalization was unremarkable, and he was discharged postoperative day two. He returned to the clinic via wheelchair two weeks later with markedly reduced swelling and pain. At this time, he was cleared to bear weight while maintaining 0 degrees flexion in the braces and was referred to physical therapy. Depending on physical therapy progress, the planned treatment protocol for the patient includes active knee extension starting three weeks post-op with the goal of full knee extension and at least 120 degrees of flexion six weeks post-op and return to sports at six months.

## Discussion

BPT rupture is an infrequent occurrence. Of the top three reasons the knee extensor mechanism can fail, unilateral patellar tendon rupture ranks behind both unilateral quadriceps tendon rupture and patellar fracture and makes up only about 2.3% of extensor mechanism injuries [6]. BPT rupture is an even rarer entity. Several meta-analyses have been conducted with patients that suffered these unique injuries. Analysis by Camarda et al. of 44 patients suffering bilateral extensor mechanism injuries (quadriceps or patellar tendon ruptures) showed that only 14% of patients with bilateral extensor mechanism injuries experienced BPT ruptures [3].

Healthy patients require a force approximately 17.5 times their body weight to cause a rupture in the patellar tendon and it is generally accepted that healthy tendons do not rupture [7]. Additionally, Kellersmann et al. conducted a review of the literature spanning from 1960 to 2003 focusing solely on patients with BPT rupture which revealed that 62% of these patients did not have systemic disease [8]. Despite a review of 50 case studies showing that 62% of patients did not have a systemic disease, most case reports in the scientific literature state that certain risk factors and conditions make one more susceptible to injury. Preexisting systemic conditions known to weaken collagen structures include rheumatoid arthritis, SLE, diabetes mellitus, hyperparathyroidism, and chronic renal disease requiring dialysis [1]. The two most common comorbidities seen with BPT rupture are SLE, where inflammatory changes are evident at the rupture site, and chronic kidney disease, which may show evidence of amyloid deposition. Individuals with these conditions are particularly susceptible to tendon rupture during non-strenuous activity [1,3,9-11]. Our patient did not have any personal or family medical history; however, he was obese with a body mass index of thirty-nine. Although the patient had not been diagnosed with diabetes mellitus at the time of his injury, it is a possibility with his BMI that he may be prediabetic or have undiagnosed diabetes mellitus. In obese patients, not only is there extra stress placed on the tendons and ligaments in the body from excess weight, but fatty degeneration of the tendons predispose to rupture similar to patients with diabetes mellitus due to an increase in advanced glycation end products [3].

Interestingly, a study by Owens et al. analyzed the number of major lower extremity tendon ruptures (quadriceps, Achilles, patellar) from a United States military treatment center. The study reported that African American armed forces services members had an approximate 4.5 times increased risk of patellar tendon rupture than white service members [12]. Although this data may not be generalizable to the general public, the patient's race may have predisposed them to this injury.

In patients without systemic disease, like our patient, patellar tendon rupture most commonly resulted from a sudden halt (i.e., stumble, abrupt stop while playing soccer, landing after jumping) while participating in a sport or other strenuous activity [8]. In comparison, patients with systemic disease were more likely performing less stressful activities like stepping onto a bus, getting up from a chair, or walking up and down stairs. Interestingly, in the study by Kellersmann et al., the average age of patients without systemic disease and BPT rupture was 42, and the average age of patients without systemic disease and BPT rupture was 36 years, both of which were significantly higher than the presented patient [8].

While controversial, corticosteroids and fluoroquinolone antibiotic effects on tendons are also reported in the literature and the medical community. Many authors report that there is a correlation between systemic ingestion of corticosteroids for chronic disease and local corticosteroid injection for symptomatic relief in sports injuries as corticosteroids are known to cause necrosis and disorganization of the fibrils weakening the collagen structures [1,13]. Interestingly, a meta-analysis of 44 patients with extensor mechanism injury showed only one patient who reported taking steroids daily [3]. However, to our knowledge, there are no presented cases of patients with SLE reporting rupture unless systemic steroids were used concomitantly. A literature review of 17 patients with SLE and BPT rupture also showed that all of these patients had been on prednisone therapy for seven to fifteen years and had other side effects due to the treatment like moon facies and osteoporosis [14]. The use of fluoroquinolones has been linked to tendinopathy in older patients (average 64 years) in the lower limbs, namely the Achilles tendon, with a minimal number of reports of bilateral rupture after use of the antibiotic [15-18]. Although controversial, our patient reported no history of systemic or injected corticosteroid use or recent antibiotic use.

Such a rare injury lends itself to patients presenting to the emergency department and ruptures being overlooked as 28%-38% of patellar tendon ruptures are initially misdiagnosed [4,19]. Some authors recommend utilizing lateral radiographs and the Insall-Salvati ratio to determine if patients do indeed have patella alta indicating a potential patellar tendon rupture. The Insall-Salvati ratio is calculated by measuring the largest diagonal length of the patella and dividing that by the length of the patellar tendon on a lateral radiograph. A ratio of less than 0.80 is indicative of patella alta and can be confirmed with magnetic resonance imaging or ultrasound studies [20].

The use of these imaging studies and prompt clinical acuity are essential to the diagnosis and prognosis of the patient's future knee function. Aforementioned, misdiagnosis and delay of surgical repair can lead to up to 19% of cases being delayed over two weeks [19]. This delay in surgical intervention can lead to excessive patellar retraction, scarring, and atrophy leading to a more complicated repair with the potential use of tendon grafts and an extended rehabilitation time that can be detrimental to a patient's prognosis [20].

## Conclusions

BPT rupture should not be overlooked in patients without known predisposing risk factors who present after minor trauma, pain, swelling, and the inability to fully extend their knee. Timely diagnosis with the use of plain radiographs and prompt surgical intervention is critical to maintaining the function of the knee joint.
